# Impact of chronic opioid on cognitive function and spermatogenesis in rat: An experimental study

**DOI:** 10.18502/ijrm.v22i7.16971

**Published:** 2024-09-12

**Authors:** Hamid Norioun, Seyed Jamal Moshtaghian, Firoozeh Alavian, Maryam Khombi Shooshtari, Golnaz Alipour, Saeedeh Ghiasvand

**Affiliations:** ^1^Medical Genetics Department, Institute of Medical Biotechnology, National Institute of Genetic Engineering and Biotechnology (NIGEB), Tehran, Iran.; ^2^Department of Plant and Animal Biology, Faculty of Biological Science and Technology, University of Isfahan, Isfahan, Iran.; ^3^Department of Biology, School of Basic Sciences, Farhangian University, Tehran, Iran.; ^4^Chronic Renal Failure Research Center, Ahvaz Jundishapur University of Medical Sciences, Ahvaz, Iran.; ^5^Microbiology Department, Medical Faculty, Kermanshah University of Medical Sciences, Kermanshah, Iran.; ^6^Department of Biology, Faculty of Sciences, University of Malayer, Malayer, Iran.

**Keywords:** Rat, Spermatogenesis, CatSper1, Tp53, Morphine, Methadone, Cognition.

## Abstract

**Background:**

Opioid analgesics like morphine and methadone are widely used for managing severe pain; however, concerns over their potential misuse and adverse effects on the brain and reproductive system are significant.

**Objective:**

We aimed to investigate their impacts on spermatogenesis and cognitive function in male Norway rats.

**Materials and Methods:**

In this experimental study, 36 male Norway rats (250–300 gr, 6 months old) were divided into 6 groups: low-dose morphine, high-dose morphine, low-dose methadone, high-dose methadone, positive control (received normal saline at 5 mg/kg), and negative control (received no treatment). Morphine and methadone were administered intraperitoneally over 30 days at doses of 3 mg/kg and 7 mg/kg, respectively. Behavioral assessments evaluated anxiety, stress, and short- and long-term memory. Sperm parameters (viability, motility, morphology), hormonal analysis (testosterone, luteinizing hormone, follicle-stimulating hormone, estradiol), and gene expressions (*Tp53, CatSper1*) were assessed.

**Results:**

A significant reduction in rat weight was observed in the high-dose morphine group (p = 0.0045), while testicular weights remained unchanged. Sperm abnormalities were observed with high doses of methadone and morphine. High-dose methadone significantly reduced offspring count (p = 0.0004). Levels of follicle-stimulating hormone, luteinizing hormone, testosterone, and estradiol varied significantly across treatment groups. Gene expression was altered in response to treatments (p < 0.05).

**Conclusion:**

Prolonged exposure to methadone and morphine resulted in memory dysfunction, chronic stress, hormonal disturbances, altered gene expression, and fertility complications. These effects were more pronounced at higher doses, highlighting the importance of careful dosage management in opioid therapy.

## 1. Introduction

Methadone and morphine, commonly prescribed narcotics for pain management, exert their effects by inhibiting pain signal transmission through the spinothalamic tract and activating pain control mechanisms in higher nerve centers (1). These opioids can impact memory, anxiety, and cognitive function, particularly with chronic usage and at high doses, leading to tolerance, dependence, and alterations in brain function (2). Reproductive system dysfunction, including decreased serum testosterone levels in men, has been reported in chronic users of methadone and morphine (3, 4). Long-term opioid use can disrupt neurotransmitter systems and neuroplasticity, affecting memory formation and cognitive function, while also posing a risk of overdose and potential neurological damage (5). Furthermore, prolonged morphine use may influence genes involved in sperm production, such as the tumor protein p53 (*Tp53* or *p53*), which plays a crucial role in spermatogenesis and male fertility (6). Dysregulation of *Tp53* can lead to impaired sperm production and quality, increasing the risk of testicular germ cell tumors and male infertility (7). Similarly, dysregulation or mutations in the sperm-associated cation channel 1 (*CatSper1*) can disrupt calcium signaling, impair sperm motility, and reduce fertilization potential, contributing to male infertility (8, 9). This study aimed to elucidate the long-term effects of methadone and morphine administration, highlighting the physiological consequences and underlying mechanisms associated with chronic opioid use.

## 2. Materials and Methods

### Medication preparation

Morphine and methadone were obtained from the Vice-Chancellor for Food and Drug Affairs at Hamadan University of Medical Sciences, Hamedan, Iran. A 10 mg/ml morphine ampoule and a 5 mg/ml methadone ampoule were used.

### Animals, grouping, and treatments

**Figure 1:**
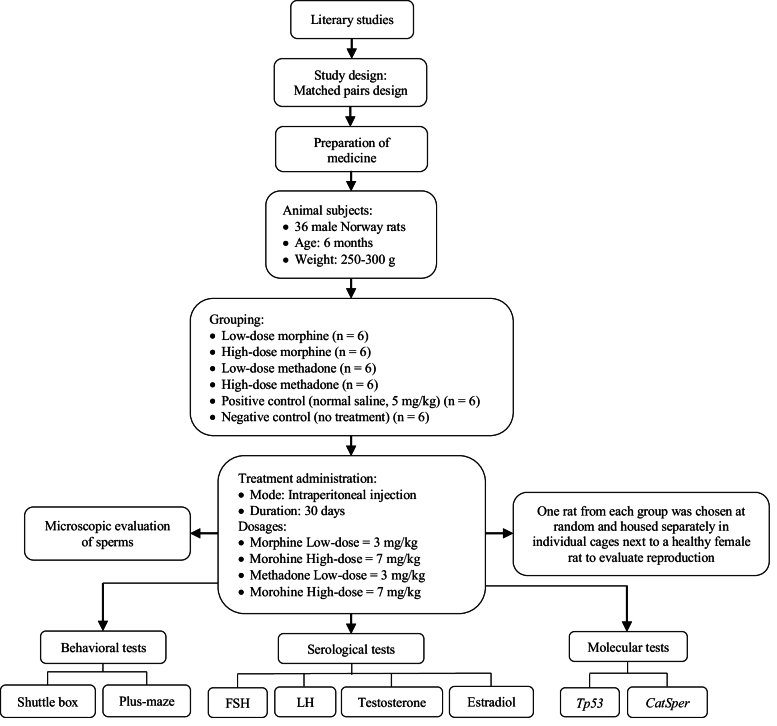
Study flow diagram.

In the current experimental study, 36 NMRI rats (6 months old, 250–300 gr), acquired from Isfahan University, Isfahan, Iran in 2022, were utilized. The rats were maintained at Isfahan University's animal facility for a month with proper access to water and nutrition, under controlled conditions (12-hr light/dark cycle, 20 C). All procedures were carried out following the laboratory animal care and use protocols (USA National Institutes of Health Publication No. 80–23, revised 1996). These rats were randomly categorized into 6 groups, each containing 6 rats. Animal tests were performed during the light phase of the 12-hr cycle. Groups 1 and 2 received morphine at 3 mg/kg and 7 mg/kg, respectively. Groups 3 and 4 received methadone at 3 mg/kg and 7 mg/kg, respectively. Group 5 received 5 mg/kg of normal saline. The duration for treatment within each cohort was set at 24 hr. All groups were treated for 30 days via subcutaneous injections. Group 6 served as a negative control with no treatment. The procedural steps of this study were formulated and executed in accordance with figure 1.

### Next-generation assessment

After completing the 30-day treatment protocol, one rat from each group was randomly selected and housed separately in individual cages with a healthy female rat to evaluate reproduction. Throughout the reproductive phase, the rats were maintained in appropriate environmental conditions, ensuring proper nutrition and regulated temperatures.

### Behavioral tests

Mental performance in Norway rats was evaluated using shuttle box and plus-maze tests following morphine and methadone treatment. The rats were acclimated to the laboratory environment for 30 min before the trials.

#### Passive avoidance test (PAT)

The PAT test was employed to evaluate rat cognitive abilities, focusing on learning and memory. The setup involved a shuttle box (BIOSEB Shuttle Boxes LE916, USA) with 2 chambers, one lit and one dark, divided by a sliding guillotine door (8 × 8 cm) and a stainless-steel rod grid floor. The dark chamber's floor was linked to a shock generator delivering electrical foot shocks (50 Hz, 1.3 mA, 3s).

During the habituation phase, each rat was placed in the well-lit chamber with the door open, allowing for free exploration for 10 min. Subsequently, in the learning trial, rats were reintroduced to the well-lit chamber, facing away from the guillotine door. After a 30 sec delay, the door was opened, and when a rat entered the dark chamber, it received a 3 sec electrical foot shock. The time taken for the rat to enter the dark chamber from when the door opened was recorded as the initial latency.

In the retention phase, occurring 24 hr after the foot shock, rats were placed in the well-lit chamber again, and the guillotine door was raised after a 30 sec delay. The time it took for a rat to enter the dark chamber was measured as the step-through latency (STL), with a maximum time limit of 300 sec. Shorter STLs indicated poorer cognitive performance, reflecting impaired memory retention (10).

#### Elevated plus maze test (EPM) 

The study utilized an EPM to assess anxiety-like behavior in rats. The EPM measured 40 cm in length, 10 cm in width, and 50 cm in height, with closed arms encircled by a 20 cm-high black wall. Each rat was initially placed in the central area of the maze, facing one of the open arms. Their behavior was observed for 10 min by a single observer who was blind to the study's details. Several behavioral parameters were recorded:

• Time spent in the open arms of the elevated EPM.

• Time spent in the closed arms of the EPM.

• Count of entries into both closed and open arms.

Anxiety levels were assessed based on the total time spent by the animals in the open arms (OAT) of the EPM and the total number of entries into the open arms (OAE). These measures were expressed as percentages:

• OAT% = (Time spent in open arms/total time spent in any arm) × 100

• OAE% = (Number of entries into open arms/total entries) × 100

The criteria for an animal entering the non-enclosed arms were the entry of the animal's hind legs. After each session, the maze was thoroughly cleaned with 70% ethanol to minimize aversive olfactory cues (11).

### Rat's weight

Before conducting any tests that necessitated sacrificing the rats, each rat's weight was measured using a Shimadzu scale (Shimadzu Corporation, Iran).

### Rat sacrifice and sample collection

At the end of the behavioral tests, the rats were anesthetized via intraperitoneal administration of ketamine hydrochloride (55 mg/kg, Sigma-Aldrich) and xylazine (11 mg/kg, Sigma-Aldrich). Testicular tissues were extracted and subsequently weighed. Carotid artery blood samples were analyzed for luteinizing hormone (LH), follicle-stimulating hormone (FSH), testosterone, and estradiol at Dr. Baradaran Medical Lab in Isfahan, Iran, using Enzyme-linked immunosorbent assay. Following World Health Organization recommendations, sperm were extracted from the head of the right testicle for physiological investigations. The sperm were incubated in Phosphate Buffered saline (Sigma-Aldrich) solution and kept at 37 C. Sperm motility and survival were assessed using an optical microscope and neobar lam. For molecular investigation, the left testicle was removed, immediately fixed in liquid nitrogen, and then stored at -70 C.

### Molecular investigations

The testicular tissue homogenization was performed using a Heidolph Homogenizer DIAX 900 (Heidolph Instruments GmbH & CO. KG, Germany). Total RNA was extracted using the SinaClon RNX_Plus extraction buffer (Sinaclon, Iran). For cDNA synthesis, a random hexamer primer was employed (TaKaRa PrimeScript II 1 ${\;}^{\text{st}}$ strand cDNA synthesis Kit, TaKaRa, Otsu, Japan; 6210B). Forward and reverse primers for *Tp53, CatSper1*, and *GAPDH* were designed using Oligo7 (V.7) and Primer3 (v. 0.4.0). The designed primers for *Tp53, CatSper1*, and *Gapdh* are listed in table I. RT-qPCR was conducted in triplicate using the Chromo 4 Bio-Rad machine (USA). *Gapdh* was selected as the calibrator gene based on previous studies and data from the Genevestigator database (https://genevestigator.com/gv/biomed.jsp).

**Table 1:** List of primers


**Gene name**	**Forward primer**	**Revers primer**	**Product size (bp)**
***Tp53***	CCTATCCGGTCAGTTGTTGGA	TTGCAGAGTGGAGGAAATGG	127
***CatSper1***	TCTTGGAGCGATGAGGAC	GACGATTGTGTTCAGGCA	204
***GAPDH***	TCTCTGCTCCTCCCTGTTCTA	ATGAAGGGGTCGTTGATGGC	177

### Ethical considerations 

The study protocol was approved by the Ethics Committee of Malayer University, Malayer, Iran (Code: IR.MALAYERU.REC.1401.004). This study was conducted in strict accordance with ethical guidelines for the use of laboratory animals. All experimental procedures involving animals were conducted in compliance with the standards set by international organizations, including the International Council for Laboratory Animal Sciences. The rights and welfare of the animals were prioritized throughout the study, ensuring that all practices adhered to the principles of humane treatment and scientific integrity.

### Statistical analysis

The *t* test was performed using GraphPad Prism 7.05 (GraphPad Software, USA) to measure and compare the mean quantification cycle (Cq) of *Tp53 *and* CatSper1* in different groups. Behavioral and hormonal tests were analyzed by one-way analysis of variance (ANOVA). P < 0.05 were considered significant. All data were reported as mean ± SD.

## 3. Results

### Behavior assessment

#### Shuttle box

**Figure 2:**
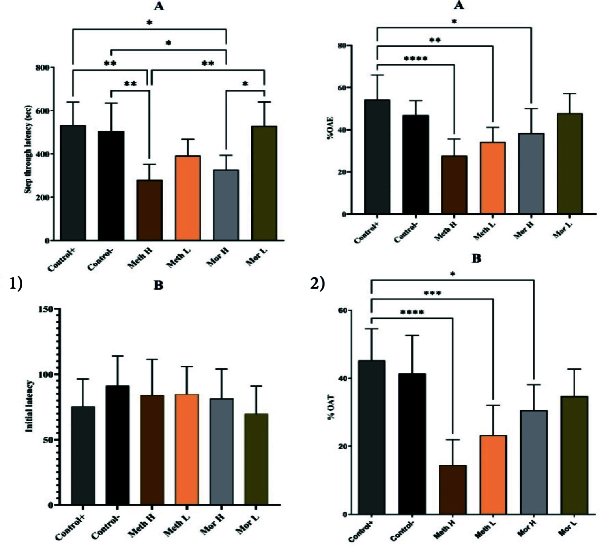
1A) Step through latency in different experimental groups during the PAT. *P < 0.05, **P < 0.01, vs control group. 1B) Initial latency: Passive avoidance test (PAT) memory of all the rats were evaluated in the shuttle box. Analyzed by one-way ANOVA followed by Tukey's post hoc test. 2A, B) The anxiety-like behaviors and locomotor activity in the elevated plus-maze test. The percentage of open arm entry (OAE) and open arm time (OAT) in all groups. *P < 0.05, **P < 0.01 and **P < 0.001 vs control group. P-values are indicated as follows: *P < 0.05, **P < 0.01, ***P < 0.001, ****P < 0.0001.

Although the rats administered low doses of methadone and morphine exhibited better performance compared to those in the high-dose methadone and morphine groups than control groups, the differences were not statistically significant (p > 0.05). However, during the retention phase, the STL in the methadone and high-dose morphine groups was significantly decreased compared to the control groups (p < 0.01 respectively, Figure 2A). The PAT memory results indicated that the initial latency was comparable across all experimental groups (p > 0.05; Figure 2B).

#### EPM

Anxiety-like behaviors and locomotor activity were assessed using EPM. Statistical analyses indicated that the percentage of time spent in the open arms (%OAT) was significantly lower in the high-dose methadone, low-dose methadone, and high-dose morphine groups when compared to the control group (p < 0.001, p < 0.01, and p < 0.05, respectively; see Figure 2A). Furthermore, the percentage of open arm entries (%OAE) was significantly reduced in both the high-dose methadone and low-dose methadone groups relative to the control group (p < 0.001 and p < 0.01, respectively; refer to Figure 2B).

### Rats weight

Rat weights showed no significant differences compared to the control groups, except for the high-dose morphine group, which experienced a notable reduction in weight compared to the positive control group (Table II, p = 0.0045).

### Sperm analysis

Sperm count did not significantly differ between the groups (p = 0.1441). However, the high-dose methadone and morphine-treated groups exhibited a notable increase in structural abnormalities, such as double-headed sperm, during microscopic examination (Table II).

### Next-generation results

After mating male rats from each experimental group with healthy females, it was observed that the group receiving a high dose of methadone had significantly fewer offspring compared to the control group. Additionally, the group treated with a high dose of morphine also had a reduced offspring count compared to the control group (Table II).

### Testicular weight

**Figure 3:**
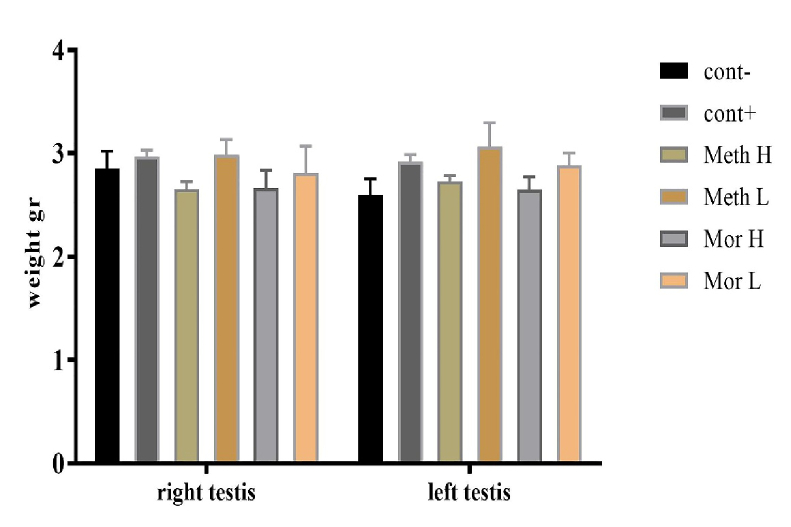
The weights of the right and left testicles in subjects treated with high and low doses of methadone and morphine did not exhibit significant changes when compared to the positive and negative control groups.

After weighing the left and right testicular tissues in each group, no significant difference was observed between them (p = 0.7027, Figure 3).

### Serology assessment

#### FSH hormone

The results of the rats' blood analysis revealed a significant increase in FSH hormone levels in rats treated with methadone at a dose of 7 mg/kg (p < 0.0001, Table III).

#### LH hormone

Unlike the FSH hormone levels, LH hormone levels increased in all groups compared to the control groups (p < 0.0001). However, the group receiving 3 mg/kg of methadone showed a smaller fold change in LH levels, whereas the group receiving 3 mg/kg of morphine displayed the highest fold change in LH levels (Table III).

#### Testosterone hormone

Testosterone hormone levels indicated that groups administered high doses of morphine, and methadone had significantly lower testosterone levels. In contrast, rats given low doses of morphine exhibited testosterone levels similar to those of the control group. Rats receiving low doses of methadone showed higher testosterone levels than the control group. Additionally, significant differences in testosterone levels were observed between the high- and low-dose groups of methadone and morphine (p < 0.0001, Table III).

#### Estradiol hormone

Estradiol hormone analysis revealed, notably, higher levels in the high-dose morphine and high-dose methadone groups, with the high-dose morphine group exhibiting the most significant departure from the normal range. In contrast, the low-dose morphine group had significantly lower estradiol levels compared to the control group, while the low-dose methadone group showed no significant change in estradiol levels compared to the control groups (p < 0.0001, Table III).

### Gene analysis

**Figure 4:**
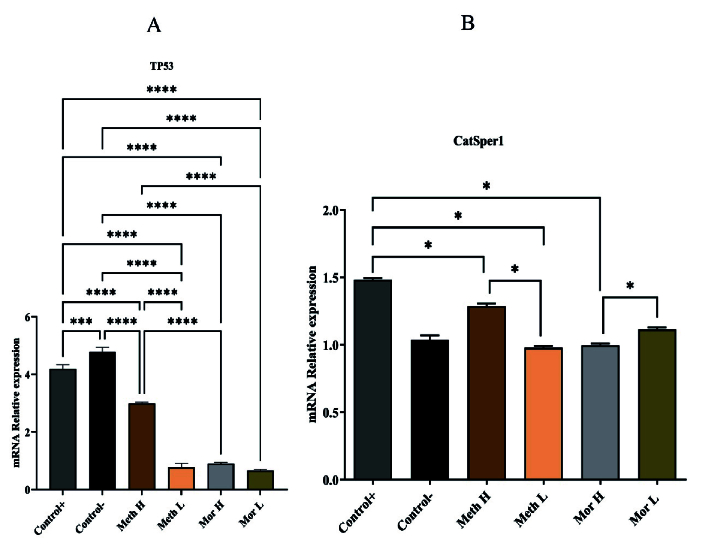
A) All treatment groups, except the high-dose methadone group, showed decreased *Tp53* gene expression compared to controls (p < 0.0001), the high-dose methadone group differed significantly from the positive control (p = 0.0003). Significant expression changes were found between the high-dose methadone group and the other treatment groups (p < 0.0001). P-values are indicated as follows: *P < 0.05, ***P < 0.001, ****P < 0.0001. B) Significant changes in *CatSper1* gene expression were observed in the low-dose methadone and high-dose morphine treatment groups compared to controls (p < 0.05). Differences were also noted between the high-dose and low-dose methadone groups (p = 0.0211), and between the high-dose and low-dose morphine groups (p = 0.0211).

*CatSper1* expression decreased in the high-dose methadone, low-dose methadone, and high-dose morphine groups compared to the control group. *Tp53* gene expression showed a significant decrease in all groups compared to the control group. Specifically, the low-dose methadone group exhibited lower *Tp53* gene expression than the high-dose methadone group. However, no significant difference was observed in *Tp53* gene expression between the high- and low-dose morphine groups (Figure 4).

**Table 2:** Summary of weight, sperm count, and birth rate changes in rats treated with methadone and morphine


**Group**	**Rat weight change (mean diff.)**	**P-value**	**Number of sperm (mean diff.)**	**P-value**	**Number of births (mean diff.)**	**P-value**
**Control ${\;}^{+}$ **	-30.8 (ns)	0.0720	36.6 (ns)	0.3138	-1.6 (ns)	0.7921
**Control ${\;}^{-}$ **	-30.8 (ns)	0.0720	-36.6 (ns)	0.3138	1.6 (ns)	0.7921
**Meth H**	-5.0 (ns)	0.9967	36.4 (ns)	0.3194	4.8 ${\;}^{**}$	0.0090
**Meth L**	-17.4 (ns)	0.5759	44.0 (ns)	0.1517	2.2 (ns)	0.5074
**Mor H**	43.6 ${\;}^{**}$	0.0045	43.6 (ns)	0.1583	3.0 (ns)	0.1952
**Mor L**	21.0 (ns)	0.3763	42.0 (ns)	0.1872	1.8 (ns)	0.7027
Weight change: The difference in weight before and after the treatment period was recorded. Number of sperm: Sperm count was measured to assess male fertility. Number of births: Recorded post-treatment to assess reproductive success. Significant findings: High-dose methadone (Meth H): Significant decrease in the number of births (4.8, p = 0.0090), high-dose morphine (Mor H): Significant increase in weight (43.6, p = 0.0045), P-value are indicated as follows: **P < 0.01, non-significant changes are marked as “ns”. Meth: Methadone, Mor: Morphine						

**Table 3:** This table presents a concise summary of significant hormonal changes observed in rats treated with varying doses of methadone and morphine compared to control groups


**Group**	**FSH (mean diff.)**	**P-value**	**LH (mean diff.)**	**P-value**	**Testosterone (mean siff.)**	**P-value**	**Estradiol (mean diff.)**	**P-value**
**Control ${\;}^{+}$ **	-0.0016 (ns)	> 0.9999	0.0006 (ns)	> 0.9999	0.25 (ns)	0.9994	-0.076 (ns)	> 0.9999
**Control ${\;}^{-}$ **	-0.0016 (ns)	> 0.9999	0.0006 (ns)	> 0.9999	0.25 (ns)	0.9994	-0.076 (ns)	> 0.9999
**Meth H**	-1.016 ${\;}^{****}$	< 0.0001	-2.091 ${\;}^{***}$	0.0001	6.96 ${\;}^{****}$	< 0.0001	-3.00 ${\;}^{**}$	0.0037
**Meth L**	-0.0028 (ns)	> 0.9999	-0.7914 (ns)	0.3090	-5.1 ${\;}^{****}$	< 0.0001	0.244 (ns)	0.9993
**Mor H**	-0.0040 (ns)	> 0.9999	-2.041 ${\;}^{***}$	0.0002	4.826 ${\;}^{****}$	< 0.0001	-4.436 ${\;}^{****}$	< 0.0001
**Mor L**	-0.0014 (ns)	> 0.9999	-2.891 ${\;}^{****}$	< 0.0001	1.368 (ns)	0.4818	5.384 ${\;}^{****}$	< 0.0001
Noteworthy findings include: High-dose methadone (Meth H) led to significant changes in FSH (-1.016, p < 0.0001), LH (-2.091, p = 0.0001), testosterone (6.96, p < 0.0001), and estradiol (-3.00, p = 0.0037). Low-dose methadone (Meth L) showed a significant decrease in testosterone levels (-5.1, p < 0.0001). High-dose morphine (Mor H) caused significant alterations in LH (-2.041, p = 0.0002), testosterone (4.826, p < 0.0001), and estradiol (-4.436, p < 0.0001). Low-dose morphine (Mor L) was associated with significant increases in LH (-2.891, p < 0.0001) and estradiol (5.384, p < 0.0001). Statistical significance: P-values are indicated as follows: **P < 0.01, ***P < 0.001, ****P < 0.0001, non-significant changes are marked as “ns”. FSH: Follicle-stimulating hormone, LH: luteinizing hormone, Meth: Methadone, Mor: Morphine								

## 4. Discussion

This study aimed to investigate the impact of methadone and morphine on memory and spermatogenesis in Norway rats. Significant weight loss occurred in the high morphine group due to metabolic disruptions, although testicular weight remained unaffected. Sperm count did not change significantly, but an increase in morphological abnormalities such as double-headed sperm, particularly in the high methadone group was observed. Rat mating experiments showed fewer offspring in rats treated with high doses of methadone and morphine.

Our study demonstrated impaired short- and long-term memory performance in rats treated with morphine and methadone, particularly in high-dose groups. This dysfunction likely results from disruptions in the mesolimbic and hippocampal pathways. Chronic use of morphine and methadone, through modulation of mu-opioid receptors, may induce receptor tolerance, adversely affecting memory and brain function. Mu-opioid receptors, integral to opioid actions including those of morphine and methadone, are part of the G protein-coupled receptor signaling pathway. These drugs primarily target mu-opioid receptors in the brain, spinal cord, and peripheral tissues, inhibiting adenylate cyclase and reducing cyclic AMP. Consequently, potassium channels are activated, hyperpolarizing neuronal membranes. This process diminishes pain signal transmission, ultimately decreasing overall neuronal activity (12). Furthermore, opioids can inhibit the release of neurotransmitters such as substance P, which transmits pain signals (13). Activation of mu-opioid receptors in reward-associated brain regions induces euphoria and relaxation, reinforcing opioid misuse. Prolonged use, as seen with morphine and methadone, leads to brain adaptations including tolerance, dependence, and alterations in neural circuitry, which promote addiction (14).

The administration of methadone and morphine can induce diverse hormonal responses in males, affecting key hormones such as FSH, LH, testosterone, and estradiol (15). Methadone therapy is associated with reduced FSH and LH levels, whereas the impact of morphine on FSH remains uncertain, with some studies suggesting potential suppression during acute administration (16). FSH plays a pivotal role in spermatogenesis by promoting the growth of Sertoli cells, stimulating spermatogonia proliferation, and facilitating the synthesis of androgen-binding protein and inhibin (17). Excessively elevated FSH levels can disrupt spermatogenesis, leading to impaired sperm development and reduced fertility. Elevated FSH may indicate hormonal imbalances within the testes or originate from pituitary gland dysfunction (18). In our study, rats subjected to prolonged high-dose methadone treatment exhibited heightened FSH levels, suggesting abnormalities in spermatogenesis. These findings underscore the potential impact of opioid use on male reproductive health.

LH is crucial in male reproductive physiology for stimulating Leydig cells in the testes to produce testosterone, which is essential for maintaining spermatogenesis. Elevated LH levels can disrupt hormonal balance, potentially leading to discrepancies between LH and FSH levels. Additionally, excessive LH stimulation may result in Leydig cells overproducing testosterone, affecting the regulatory feedback loop for testosterone secretion, and influencing sperm production (19). Elevated LH levels observed across all study groups may consequently impair sperm quality, reduce fertility, cause hormonal imbalances, and potentially lead to testicular dysfunction.

Testosterone plays a crucial role in stimulating seminiferous tubules in the testes, which is essential for sperm development and maturation. Low testosterone levels can hinder this process, reducing sperm quantity and potentially leading to infertility (20). Additionally, testosterone significantly influences sperm quality by affecting morphology, motility, and fertility. Decreased testosterone levels may result in abnormal sperm morphology, decreased motility, and impaired fertilization. Beyond reproduction, testosterone governs sexual function and libido; reduced levels can lead to decreased sexual desire and erectile dysfunction, which can affect fertility (21).

High doses of morphine and methadone notably decreased testosterone levels, while low doses of methadone increased it; low doses of morphine did not show a significant change. Furthermore, these drugs were associated with reduced sperm count, increased abnormal sperm morphology, diminished sexual drive, and fewer offspring. These findings are consistent with previous research, where linking decreased testosterone levels to sperm abnormalities, altered mating behavior, and fertility outcomes.

In males, estradiol is produced from testosterone through aromatization, primarily occurring in the testes, adipose tissue, and brain (22). Despite lower levels of estradiol in males compared to females, its presence plays a significant role in various aspects of male reproductive function. Both low and excessively high levels of estradiol can disrupt spermatogenesis. Estradiol is a vital component of the intricate hypothalamus-pituitary-testicular axis, regulating testosterone and sperm production. It interacts with the hypothalamus and pituitary gland, influencing their communication with the testes and potentially affecting hormone production and sperm development. Estradiol receptors are found in various segments of the male reproductive tract, including the prostate gland and epididymis, indicating its role in critical functions such as sperm maturation, storage, and transit.

Maintaining a delicate balance between estradiol and testosterone is essential, as these hormones mutually influence each other's synthesis. Excessive estradiol relative to testosterone can lead to estrogen dominance, disrupting the necessary hormonal environment for regular sperm production (23).

In our study, groups treated with high doses of methadone and morphine exhibited significantly increased estradiol levels compared to the control group, while the low-dose morphine group showed decreased levels. Elevated estradiol levels could potentially explain the observed decline in testosterone levels among the rats and may indirectly influence the sexual behavior of the study participants.

Extended exposure to opioids, including substances like morphine and methadone, may influence the *Tp53* gene. Opioids are associated with cellular stress and oxidative damage, potentially leading to misregulation of *Tp53* (24). *Tp53* plays a crucial role in spermatogenesis and regulation of apoptosis. Impaired *Tp53* function compromises apoptosis, affecting sperm quality and fertility (25). Our study revealed a significant reduction in *Tp53* expression in rats exposed to morphine and methadone, indicating potential fertility issues due to genetic anomalies and disrupted spermatogenesis.

*CatSper1* is a protein integral to male reproductive health, forming a crucial component of the CatSper ion channel complex located predominantly in the sperm tail. This complex governs the influx of calcium ions into sperm, essential for various sperm functions including motility, capacitation, and the acrosome reaction. Disruptions in *CatSper1* regulation can lead to male infertility, affecting critical aspects of sperm physiology (26).

Firstly, *CatSper1* deficiency impairs sperm motility, as calcium ions are necessary for the whip-like movement of the sperm tail, crucial for efficient navigation and reaching the egg for fertilization. Secondly, it hinders capacitation, a process where sperm undergoes physiological changes to become capable of fertilizing an egg, mediated by *CatSper1*-mediated calcium signaling. Lastly, *CatSper1* deficiency can impede the acrosome reaction, a crucial step in fertilization involving the release of enzymes enabling sperm to penetrate the protective layers surrounding the egg (27). Furthermore, mutations or irregularities in the *CatSper1* gene or ion channel complex can compromise sperm motility and reduce fertility, posing challenges to natural conception (28).

In our study, downregulated *CatSper1* expression in morphine and methadone-treated rats suggests potential fertility issues due to altered ion channel function, essential for sperm survival and fertility within the female reproductive system.

## 5. Conclusion

Prolonged use of morphine and methadone can significantly impair cognitive function by affecting cortical opioid receptors. These substances also disrupt spermatogenesis, impacting testicular tissue receptors and hormone synthesis, including LH, FSH, estradiol, and testosterone. Furthermore, they may influence genes involved in sperm development, such as *Tp53* and *CatSper1*, potentially causing dysfunction.

The current investigation demonstrated that the administration of methadone and morphine across varying dosages elicits dose-dependent reactions.

##  Data availability

Data supporting the findings of this study are available upon reasonable request from the corresponding author.

##  Author contributions

H Norioun and S Ghiasvand had full access to all of the data in the study and took responsibility for the integrity of the data and the accuracy of the data analysis. Concept and design: H Norioun, S.J Moshtaghian, M Khombi Shooshtari, and S Ghiasvand. Acquisition, analysis, or interpretation of data: H Norioun, S.J Moshtaghian, M Khombi Shooshtari, F Alavian, and S Ghiasvand. Product preparation: H Norioun, G Alipour, and S Ghiasvand. Drafting of the manuscript: H Norioun, M Khombi Shooshtari, G Alipour, and S Ghiasvand. Critical revision of the manuscript for important intellectual content: H Norioun and S Ghiasvand. Statistical analysis: H Norioun and M Khombi Shooshtari. Supervision: S Ghiasvand.

##  Conflict of Interest

The authors declare that there is no conflict of interest.

## References

[bib1] Kreutzwiser D, Tawfic QA (2020). Methadone for pain management: A pharmacotherapeutic review. CNS Drugs.

[bib2] Badshah I, Anwar M, Murtaza B, Khan MI (2023). Molecular mechanisms of morphine tolerance and dependence; novel insights and future perspectives. Mol Cell Biochem.

[bib3] Ghasemi-Esmailabad S, Talebi AH, Talebi AR, Amiri S, Moshrefi M, Pourentezari M (2022). The effects of morphine abuse on sperm parameters, chromatin integrity and apoptosis in men. JBRA Assist Reprod.

[bib4] Ortman HA, Siegel JA (2020). The effect of methadone on the hypothalamic pituitary gonadal axis and sexual function: A systematic review. Drug Alcohol Depend.

[bib5] Thompson BL, Oscar-Berman M, Kaplan GB (2021). Opioid-induced structural and functional plasticity of medium-spiny neurons in the nucleus accumbens. Neurosci Biobehav Rev.

[bib6] Asgharzadeh F, Roshan-Milani S, Fard AA, Ahmadi K, Saboory E, Pourjabali M, et al (2021). The protective effect of zinc on morphine-induced testicular toxicity via p53 and Akt pathways: An in vitro and in vivo approach. J Trace Elem Med Biol.

[bib7] Jaiswal D, Trivedi S, Agrawal NK, Singh K (2015). Dysregulation of apoptotic pathway candidate genes and proteins in infertile azoospermia patients. Fertil Steril.

[bib8] Brown SG, Publicover SJ, Barratt CL, Martins da Silva SJ (2019). Human sperm ion channel (dys) function: Implications for fertilization. Hum Reprod Update.

[bib9] Heidary Z, Zaki-Dizaji M, Saliminejad K, Khorramkhorshid HR (2019). Expression analysis of the CRISP2, CATSPER1, PATE1 and SEMG1 in the sperm of men with idiopathic asthenozoospermia. J Reprod Infertil.

[bib10] Khombi Shooshtari M, Sarkaki A, Mansouri SMT, Badavi M, Khorsandi L, Ghasemi Dehcheshmeh M, et al (2020). Protective effects of Chrysin against memory impairment, cerebral hyperemia and oxidative stress after cerebral hypoperfusion and reperfusion in rats. Metab Brain Dis.

[bib11] Hajipour S, Shooshtari MK, Farbood Y, Mard SA, Sarkaki A, Chameh HM, et al (2023). Fingolimod administration following hypoxia induced neonatal seizure can restore impaired long-term potentiation and memory performance in adult rats. Neuroscience.

[bib12] Stoveken HM, Zucca S, Masuho I, Wang D, Dao M, Grill B, et al (2020). GPR139 signals through Gq/11 to oppose mu opioid receptor signaling. J Biol Chem.

[bib13] Dou B, Li Y, Ma J, Xu Z, Fan W, Tian L, et al (2021). Role of neuroimmune crosstalk in mediating the anti-inflammatory and analgesic effects of acupuncture on inflammatory pain. Front Neurosci.

[bib14] Zhang H, Largent-Milnes TM, Vanderah TW (2020). Glial neuroimmune signaling in opioid reward. Brain Res Bullet.

[bib15] Haddadi M, Ai J, Shirian S, Kadivar A, Farahmandfar M (2020). The effect of methadone, buprenorphine, and shift of methadone to buprenorphine on sperm parameters and antioxidant activity in a male rat model. Comp Clin Pathol.

[bib16] Mohammadi N, Shirian S, Gorji A, Roshanpajouh M, Ahmadi E, Nazari H, et al (2023). The potential protective effect of melatonin and N-acetylcysteine alone and in combination on opioid-induced testicular dysfunction and degeneration in rat. Reprod Toxicol.

[bib17] Oduwole OO, Peltoketo H, Huhtaniemi IT (2018). Role of follicle-stimulating hormone in spermatogenesis. Front Endocrinol.

[bib18] Santi D, Crépieux P, Reiter E, Spaggiari G, Brigante G, Casarini L, et al (2020). Follicle-stimulating hormone (FSH) action on spermatogenesis: A focus on physiological and therapeutic roles. J Clin Med.

[bib19] Chao H-H, Zhang Y, Dong P-Y, Gurunathan S, Zhang X-F (2023). Comprehensive review on the positive and negative effects of various important regulators on male spermatogenesis and fertility. Front Nutr.

[bib20] Widhiantara IG, Permatasari AA, Rosiana IW, Wiradana PA, Satriyasa B (2021). Steroidogenesis mechanism, disruption factor, gene function, and role in male fertility: A mini review. Indian J Forens Med Toxicol.

[bib21] Fujisawa Y, Ono H, Konno A, Yao I, Itoh H, Baba T, et al (2022). Intrauterine hyponutrition reduces fetal testosterone production and postnatal sperm count in the mouse. J Endocr Soc.

[bib22] Gurung P, Yetiskul E, Jialal I (2022). Physiology, male reproductive system.

[bib23] Schulster M, Bernie AM, Ramasamy R (2016). The role of estradiol in male reproductive function. Asian J Androl.

[bib24] Gilardi F, Augsburger M, Thomas A

[bib25] Nguyen DH, Soygur B, Peng S-P, Malki S, Hu G, Laird DJ (2020). Apoptosis in the fetal testis eliminates developmentally defective germ cell clones. Nat Cell Biol.

[bib26] Shanaz S, Hamadani A, Firdous S, Shah R, Rather MA, Khan NN, et al (2022). CatSper genes and their role in male infertility: A review. SKUAST J Res.

[bib27] Lishko PV, Mannowetz N (2018). CatSper: A unique calcium channel of the sperm flagellum. Curr Opin Physiol.

[bib28] Wang H, McGoldrick LL, Chung J-J (2021). Sperm ion channels and transporters in male fertility and infertility. Nat Rev Urol.

