# Corrigendum to “Impact of chronic opioid on cognitive function and spermatogenesis in rat: An experimental study” [Int J Reprod BioMed 2024; 22: 579-592]

**DOI:** 10.18502/ijrm.v22i10.17671

**Published:** 2024-12-02

**Authors:** Hamid Norioun, Seyed Jamal Moshtaghian, Firoozeh Alavian, Maryam Khombi Shooshtari, Golnaz Alipour, Saeedeh Ghiasvand

The publisher has been informed of an error that occurred on page 579 in which the last authors affiliation must be changed to Department of Biology, Faculty of Sciences, Malayer University, Malayer, Iran. On behalf of the author, the publisher wishes to apologize for this error. The online version of the article has been updated on October 31, 2024 and can be found at https://doi.org/10.18502/ijrm.v22i7.16971.

